# Some Data on the Distribution of the Milk Factor

**DOI:** 10.1038/bjc.1949.56

**Published:** 1949-12

**Authors:** L. Dmochowski


### SOME DATA ON THE DISTRIBUTION OF THE MILK FACTOR.

L. DMOCHOWSKI.

*From the Department of Experimental Pathology and Cancer Research, Medical School, University of Leeds.*

Received for publication October 26, 1949.

AFTER the discovery of the mammary tumour agent or the milk factor in the milk of high-breast-cancer strain female mice (Bittner, 1936), numerous investigations were undertaken in order to ascertain its presence in various tissues and organs of normal mice of high-breast-cancer strains, and also in spontaneous breast tumours of these mice. For this purpose either implantation of various organs into suitable susceptible test mice was used, or extracts of these organs and tissues were made and injected into the test mice. In this way, on the basis of the development of breast cancer in the test mice, the milk factor was found to be present in the spleen (Bittner, 1939*a* ; Andervont,

Shimkin and Bryan, 1942 ; Dmochowski, 1944*a*), thymus (Bittner, 1939*a*, *b*), lactating mammary tissue (Bittner, 1939*a* ; Andervont, Shimkin and Bryan, 1942 ; Andervont and Bryan, 1944), spontaneous breast tumour tissue (Bittner, 1941*a* ; Bryan, Kahler, Shimkin and Andervont, 1942 ; Visscher, Green and Bittner, 1942 ; Andervont and Bryan, 1944 ; Dmochowski, 1944*b* ; Barnum, Ball and Bittner, 1947), transplanted breast tumour tissue (Barnum, Ball and Bittner, 1947 ; Dmochowski, 1949), Harderian gland (Bittner and Watson, 1946). The factor is also present in whole blood (Woolley, Law and Little, 1941), and is more concentrated in cellular elements than in the serum (Bittner, 1945*a* ; Hummel and Little, 1949). The presence of the factor in some organs such as liver was doubtful (Bittner, 1941*b*, 1947), or was not ascertained as in the stomach milk, and there existed contradictory evidence about the transmission of the milk factor in the uterus of high-breast-cancer strain females (Fekete and Little, 1942 ; Andervont, 1945). All organs and tissues tested were those of high-cancer strain females, and it was only after the present experiments had been started that Andervont and Dunn (1948) reported the presence of the milk factor in the seminal vesicles of high-breast-cancer strain males, and quite recently Hummel and Little (1949) found the factor in the blood of high-cancer strain males.

In order to control some of the results of electron microscope studies of normal tissues of high- and low-breast-cancer strain mice, first reported by Passey, Dmochowski, Astbury and Reed in 1947, experiments were commenced during the second half of 1947, in order to ascertain the presence or the absence of the milk factor in : (1) normal organs of C3H high-breast-cancer strain males, (2) liver of C3H high-breast-cancer strain female mice, (3) normal organs of IF low-breast-cancer strain mice, (4) placenta and embryos of C3H high-cancer strain mice. In May, 1948, as the result of the electron microscope findings of large numbers of the characteristic particles in stomach milk of C3H mice (Passey, Dmochowski, Astbury and Reed, 1947–1948*a* ; Passey, Dmochowski, Astbury, Reed and Johnson, 1948), an additional experiment was carried out to test the stomach milk of 5-day-old C3H high-cancer strain mice to ascertain if the particles could be associated with the presence of the factor.

### *Experiment No.* 1.

This experiment was carried out in order to give additional information about the results of electron microscope examinations of extracts of normal organs of high-breast-cancer strain mice in which, as in breast tumours of these mice, spherical particles presumed to be the milk factor itself had been described (Passey, Dmochowski, Astbury and Reed, 1947 ; 1947–1948*b*). Preliminary results of this experiment have already been reported (Dmochowski, 1947–1948, 1948*a*, 1948–1949*a*), and a full account is now presented.

#### *Methods.*

As the source of material the following organs of C3H high-breast-cancer strain males, 2–4 weeks old, were used : lungs, kidneys, spleen, thymus and heart. The organs from 14 C3H strain males were minced together, desiccated in the usual way (Dmochowski, 1946), and after a storage in the ice-chest for a fortnight used in the experiment. The desiccated organs were resuspended in distilled water in a proportion of 1:10, and injected subcutaneously every second day in 0·5 c.c. quantities into the test mice. Each injection contained an amount of dried tissue equal to 0·05 g. of fresh tissue. Each mouse received 22 injections, corresponding to a total of 1·1 g. of fresh tissue. Eighteen C57 × R III susceptible hybrid females, 4–6 weeks old, were used. All females in this, as well as in the other experiments described, were forcibly bred by removing the first three litters within 24 hours of birth, and then allowed to breed in a normal way and kept under similar conditions on a diet of "rat-cake" cubes and oats, with an unlimited supply of tap water.

*Results.*

The results of the experiment are summarized in Table I. As can be seen from Table I, repeated administration of dried normal organs of 2- to 4-weeks-old C3H high-breast-cancer strain males induced breast tumours in 6 out of 15 susceptible test mice alive at the appearance of the first tumour.

*Discussion.*

It is not known how far the use of several organs of C3H high-cancer strain males, mixed and dried together, has influenced the results. There is no doubt, however, that C3H high-breast-cancer strain males possess the milk factor. Similar results have recently been reported by Andervont and Dunn (1948), who have demonstrated the presence of the factor in the seminal vesicles of C3H high-cancer strain males, maintained in their laboratories, and by Hummel and Little (1949), who have found the factor in the whole blood of dba high-breast-cancer strain males. It can be concluded that the milk factor is present not only in females but also in males of high-breast-cancer strains.

*Experiment No. 2.*

*Methods.*

Livers of 18 C3H high-breast-cancer strain females, 2–4 weeks old, were used as the source of material. The liver tissue was minced together, desiccated, and after one week's storage in the ice-chest, ground with distilled water in a proportion of 1:10, and injected subcutaneously every second day in 0·5 c.c. quantities into 15 4- to 6-weeks-old C57 × R III hybrid females. Each of the mice received 22 injections, giving an amount of dried material equal to 1·1 g. of fresh liver tissue.

*Results.*

The results are shown in Table II. Repeated injections of dried liver tissue of C3H high-breast-cancer strain females induced breast cancer in 2 out of 18 test mice alive at the appearance of the earliest tumour.

*Discussion.*

In previous experiments the method of repeated injections (Dmochowski, 1945) was found to be very effective. Therefore, in view of the large amount of material injected, it can be stated that the milk factor is present in the liver of young female mice of C3H high-cancer strain, but it is a matter of speculation whether it is present in small quantities or in an attenuated form. Hummel and

Little (1949) in their recently published experiments described a similar incidence of breast cancer in test mice following subcutaneous implantation of liver tissue or subcutaneous injections of minced liver tissue suspended in distilled water. They have put forward a suggestion that liver may carry the milk factor only by virtue of the blood it contains. The presence of small amounts of the factor in the liver tissue, at first not revealed in Bittner's experiments (1941*b*), will have to be further investigated in order to elucidate what part, if any, is played by the liver in the removal and destruction of the mammary tumour agent.

### *Experiment No.* 3.

In connection with the presence of the milk factor in various tissues and organs of high-breast-cancer strain female mice, and the investigations of the presence of the agent in breast tumours induced in IF low-breast-cancer strain females after treatment with methylcholanthrene (Dmochowski and Orr, 1949), experiments were carried out to test normal organs of IF low-cancer strain females for the presence of the milk factor.

*Methods.*

The following organs of IF low-cancer strain females, 2–4 weeks old, were used as the source of material : kidneys, spleen, heart, lungs and thymus. These organs were minced together, desiccated, and stored for a fortnight in the ice-chest. Twelve C57 × RIII hybrid females, 4 weeks old, each were given 12 subcutaneous injections of a distilled water suspension of the dried organs. Altogether a total amount of material equal to 1·15 g. of fresh tissue was injected into each mouse.

*Results.*

Table III gives a summary of the results. No tumours developed in any of the test mice, although all of them survived beyond the age of appearance of the earliest tumours in the two previous experiments.

*Discussion.*

The results, taken together with the results of the tests for the presence of the milk factor in breast tumours induced in IF strain females following the application of methylcholanthrene (Dmochowski and Orr, 1949), give a fair indication that IF strain females do not harbour the milk factor. During the same interval of time breast tumours were obtained in test mice injected with similar quantities of dried normal organs and liver of C3H strain mice. This again points towards a conclusion that the factor is not present in the organs of IF low-breast-cancer strain females, at least in quantities which could be detected under the conditions of the test employed. Hummel and Little (1949) have reported similar results with the blood, liver and spleen of their D low-breast-cancer strain female mice.

### *Experiment No.* 4.

Fekete and Little (1942) reported intra-uterine transmission of the milk factor by reciprocal transference of fertilized ova between female mice of high- and low-breast-cancer strains. Mice born from the transferred ova were nursed by mothers which gave birth to them. The original C57 low-cancer strain mice born from transferred ova developed an incidence of 50 per cent breast tumours and three generations of their descendants gave an incidence of 73 per cent. The original dba high-cancer strain mice born from transferred ova had no tumours, while their progeny showed an incidence of 12 per cent breast cancer. Fekete and Little (1942) suggested that the intra-uterine transmission may have been responsible for their results. However, Bittner (1945*b*) reported an incidence of 1·4 per cent in 20 generations of foster nursed Strong A high-breast-cancer strain mice, and an incidence of 1·9 per cent in 10 generations of foster nursed C3H high-breast-cancer strain females. Andervont (1945) observed no breast tumours in 5 generations of mice descended from C3H young mice removed from the uterus of C3H high-cancer strain mothers and foster nursed by C57 low-cancer strain females. Should an intra-uterine transmission of the milk factor take place, as suggested by Fekete and Little (1942), the incidence of breast tumours in Bittner's experiments would have been much higher and Andervont would have observed breast tumours in mice of his experiments. The high incidence of breast tumours in the C57 low-cancer strain mice of Fekete and Little may be partially explained by the suckling of high-cancer strain mothers and also by the action of hormonal factors. The development of breast tumours in the dba strain mice is, however, more difficult to explain and requires further investigation. In connection with these contradictory observations an experiment was carried out in an attempt to elucidate this problem from a different angle.

*Methods.*

C3H high-breast-cancer strain embryos and placentas served as the source of material. They were removed from the uterus of several C3H high-cancer strain females in the last few days of pregnancy, and care was taken to wash off with distilled water all traces of maternal blood from the embryos, carefully separated from their placentas. All the embryos were then minced together and desiccated. Similarly all the placentas were minced and dried together. C57 × RIII hybrid females, 4–6 weeks old, mostly litter mates, were divided into two groups, each comprising 18 animals. Each mouse of the first group received 14 subcutaneous injections of a 1 : 10 distilled water suspension of desiccated embryos, giving a total equal to 0·7 g. of fresh tissue. Each mouse of the second group received a similar number of subcutaneous injections of a 1 : 10 distilled water suspension of dried placenta, giving a total of dried tissue corresponding to 0·7 g. of fresh tissue.

*Results.*

The results of the experiment are shown in Table IV. No breast tumours developed in any of the mice injected with dried embryos' tissue, in spite of the large quantities of the tissue injected, and only 2 breast tumours developed in test mice injected with dried placenta.

*Discussion.*

Hummel and Little (1949), in their recently published experiments, observed no tumours in test mice injected with either placentas of C3H high-cancer strain females or with the blood of their foetuses. The difference between their results and the present observation of 2 breast tumours in test mice injected with dried placentas of C3H high-cancer strain mice may be due to the large quantities of tissue injected. The results permit the conclusion that the milk factor is not present in C3H high-cancer strain embryos before their birth and is present, perhaps only in very small quantities, in the placenta of C3H high-cancer strain females. The method of repeated injections (Dmochowski, 1945) should have revealed the presence of small amounts of the milk factor in the embryos, as it has revealed it in the liver tissue or in the placentas. It is not known whether this is due to some unknown factor or factors which neutralize the milk factor in the placenta. Hummel, Little and Eddy (1949) have published results which seem to indicate that the mature placenta is the site of the neutralization or inhibition of the milk factor. It seems that there is no intra-uterine transmission of the milk factor, and the present results corroborate the findings of Bittner (1945b), and of Andervont (1945), as well as the recent observations by Hummel and Little (1949).

*Experiment No.* 5.

Electron microscope examination of extracts of milk obtained from stomachs of 5-day-old C3H offspring had revealed the presence of the same spherical particles (Passey, Dmochowski, Astbury, Reed and Johnson, 1948) as those which had been observed in extracts of normal and malignant tissues of mice of several high-breast-cancer strains. Biological tests were, therefore, carried out to ascertain whether the stomach milk containing these particles possesses the breast tumour inducing property. Preliminary results of these tests, already briefly reported (Dmochowski, 1948*b*, 1948–1949*b*), are now described in detail.

*Methods.*

In a further search for a good supply of the milk factor, stomach milk of 5-day-old C3H high-breast-cancer strain mice was examined. The stomach milk was obtained from young C3H high-cancer strain mice by killing the animals under ether anaesthesia half-an-hour after they suckled their mothers, and was desiccated in the usual manner. The test mice, 4–6 weeks old, were divided into 2 groups. One group received 10 subcutaneous injections of a 1 : 10 distilled water suspension of desiccated stomach milk, equal to a total of 0·95 g. of fresh milk, the other group received 1 injection of a similar suspension of the stomach milk, corresponding to 0·1 g. of fresh milk.

*Results.*

Table V summarizes the results so far obtained. As can be seen, a higher number of breast tumours developed in the test mice given repeated doses of the stomach milk as compared with the number of tumours in animals injected with a single dose of the desiccated stomach milk. Also the breast tumours developed at a later age in the latter group of test mice than in the former group.

*Discussion.*

The present results show that the milk factor is present in the stomach milk of young 5-day-old C3H high-breast-cancer strain mice, which is therefore a good source of the mammary tumour agent. Similar results have been reported by Hummel and Little (1949),who found the stomach contents of 4- to 11-day-old Strong A and C3H strain mice a source of an active mammary tumour agent.

SUMMARY.

1. Normal organs, such as liver, lungs, spleen, kidneys, and heart of 2- to 4-weeks-old C3H high-breast-cancer strain males contain the milk factor as shown by the development of breast tumours in susceptible C57 × R III hybrid females, injected with extracts of the desiccated organs of these animals.

2. The mammary tumour agent is present only in small amounts in the liver of 2- to 4-weeks-old C3H high-cancer strain females.

3. Normal organs of young IF low-breast-cancer strain females do not harbour the milk factor. Breast tumours were not observed in test mice given repeated injections of extracts of these organs.

4. While embryos of C3H high-cancer strain female mice do not show the presence of the factor, placenta of these mice contains the milk factor only in very small amounts.

5. Stomach milk of 5-day-old C3H high-cancer strain mice has been found to be a good source of the mammary tumour agent.

REFERENCES.

ANDERVONT, H. B.—(1945) " Research Conference on Cancer," *Amer. Ass. Adv. Sci.*, p. 97.

*Idem* AND BRYAN, W. R.—(1944) *J. nat. Cancer Inst.*, **5**, 143.

*Idem* AND DUNN, TH. B.—(1948) *Ibid.*, **8**, 227.

*Idem*, SHIMKIN, M. B., AND BRYAN, W. R.—(1942) *Ibid.*, **3**, 309.

BARNUM, C. P., BALL, Z. B., AND BITTNER, J. J.—(1947) *Cancer Res.*, **7**, 522.

BITTNER, J. J.—(1936) *Science*, **84**, 162.—(1939*a*) *Publ. Hlth. Rep., Wash.*, **54**, 1827.—(1939*b*) *Amer. J. Cancer*, **35**, 90.—(1941*a*) *Science*, **93**, 527.—(1941*b*) *Trans. Studies Coll. Phys. Phila.*, **9**, 129.—(1945*a*) *Proc. Soc. exp. Biol. N.Y.*, **59**, 43.—(1945*b*) " Research Conference on Cancer," *Amer. Ass. Adv. Sci.*, p. 63.—(1947) *Ann. N.Y. Acad. Sci.*, **49**, 69.

*Idem* AND WATSON, C. J.—(1946) *Cancer Res.*, **6**, 337.

BRYAN, W. R., KAHLER, H., SHIMKIN, M. B., AND ANDERVONT, H. B.—(1942) *J. nat. Cancer Inst.*, **2**, 451.

DMOCHOWSKI, L.—(1944*a*) *Brit. J. exp. Path.*, **25**, 119.—(1944*b*) *Ibid.*, **25**, 138.—(1945) *Ibid.*, **26**, 192.—(1946) *Ibid.*, **27**, 391.—(1947–48) " Report of the Dept. of Exp. Path. and Cancer Res., University of Leeds," in 22nd *Annual Report of the Yorkshire Council*, British Empire Cancer Campaign, p. 8.—(1948*a*) *Ann. Rep.*, Brit. Emp. Cancer Campgn., **26**, 139.—(1948*b*) *Ibid.*, p. 137.—(1948–49*a*) "Report of the Dept. of Exp. Path. and Cancer Res., University of Leeds," in 23*rd Annual Report of the Yorkshire Council*, British Empire Cancer Campaign, p. 7.—(1948–49*b*) *Ibid.*, p. 6.—(1949) *Brit. J. Cancer*, **3**, 246.

*Idem* AND ORR, J. W.—(1949) *Ibid.*, **3**, 520.

FEKETE, E., AND LITTLE, C. C.—(1942) *Cancer Res.*, **2**, 525.

HUMMEL, K. P., AND LITTLE, C. C.—(1949) *Ibid.*, **9**, 129.

*Iidem* AND EDDY, M. S.—(1949) *Ibid.*, **9**, 135.

PASSEY, R. D., DMOCHOWSKI, L., ASTBURY, W. T., AND REED, R.—(1947) *Nature*, **160**, 565.—(1947–48*a*) " Report of the Dept. of Exp. Path. and Cancer Res., University of Leeds, in 22*nd Annual Report of the Yorkshire Council*, British Empire Cancer Campaign, p. 8.—(1947–48*b*) *Ibid.*, p. 7.

*Iidem* AND JOHNSON, P.—(1948) *Nature*, **161**, 759.

VISSCHER, M. B., GREEN, R. G., AND BITTNER, J. J.—(1942) *Proc. Soc. exp. Biol. N.Y.*, **49**, 94.

WOOLLEY, G. W., LAW, L. W., AND LITTLE, C. C.—(1941) *Cancer Res.*, **1**, 955.


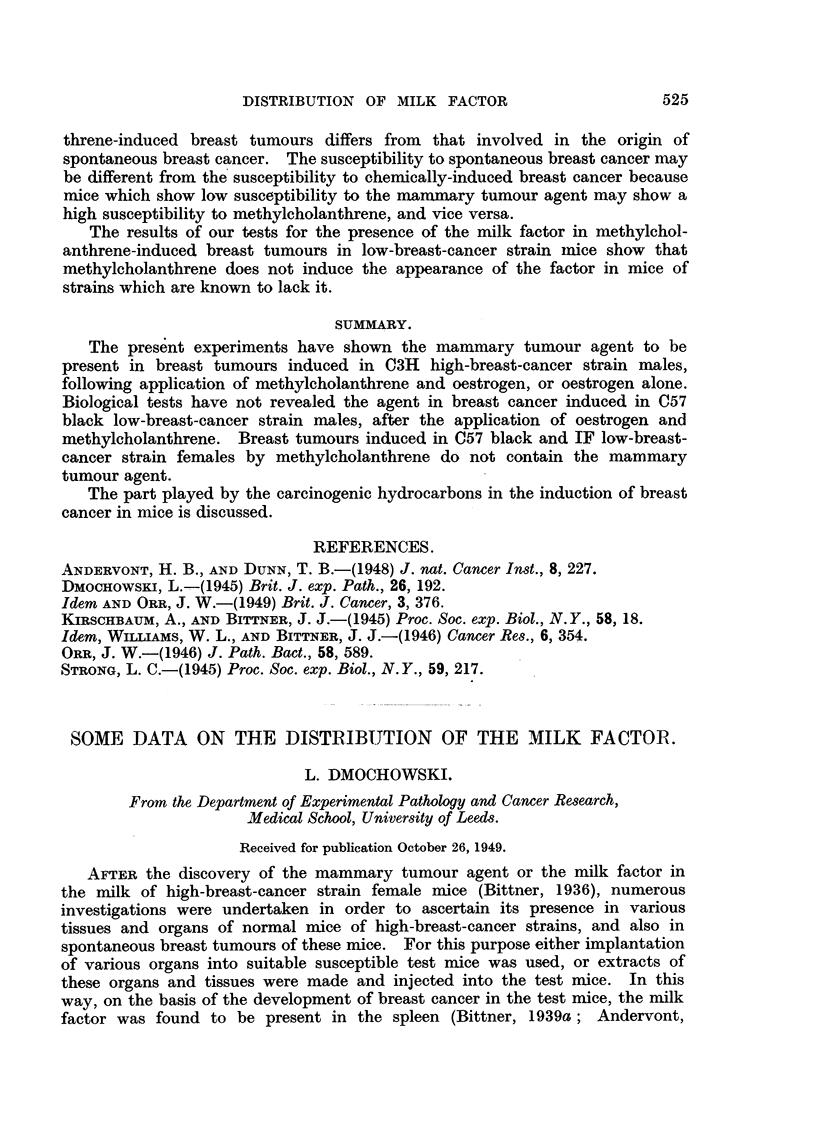

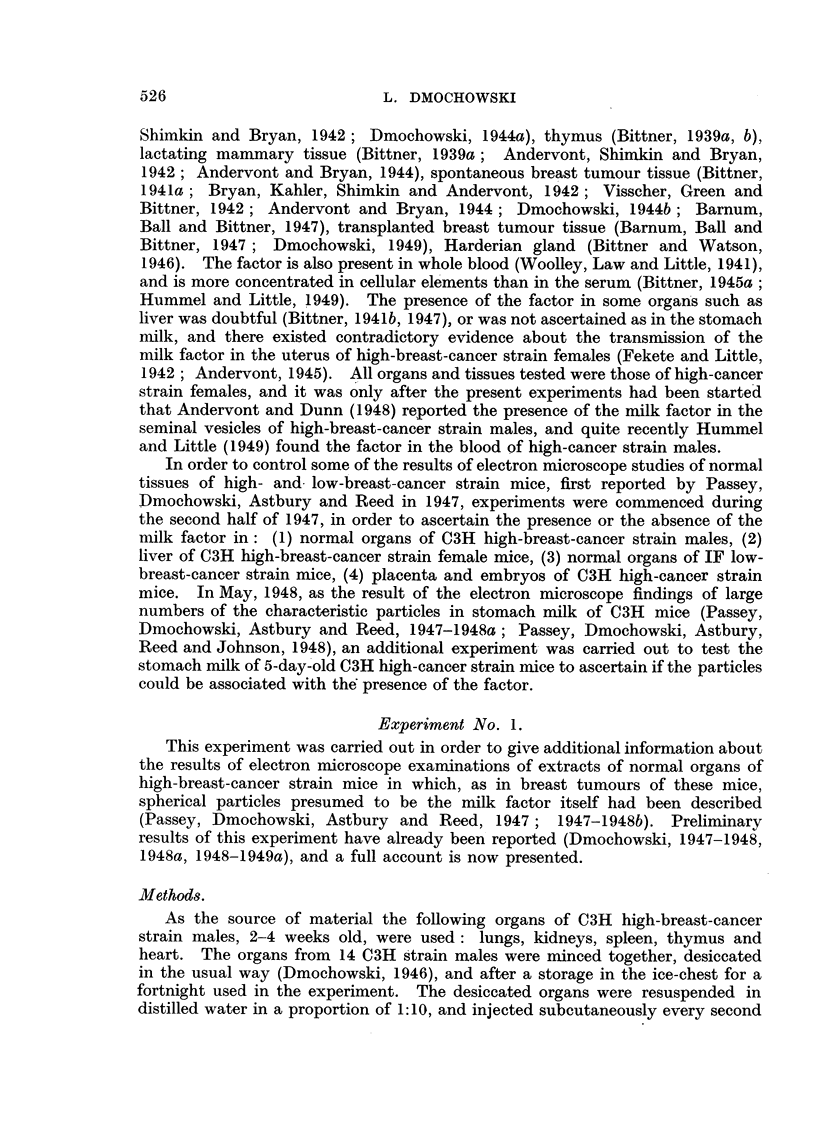

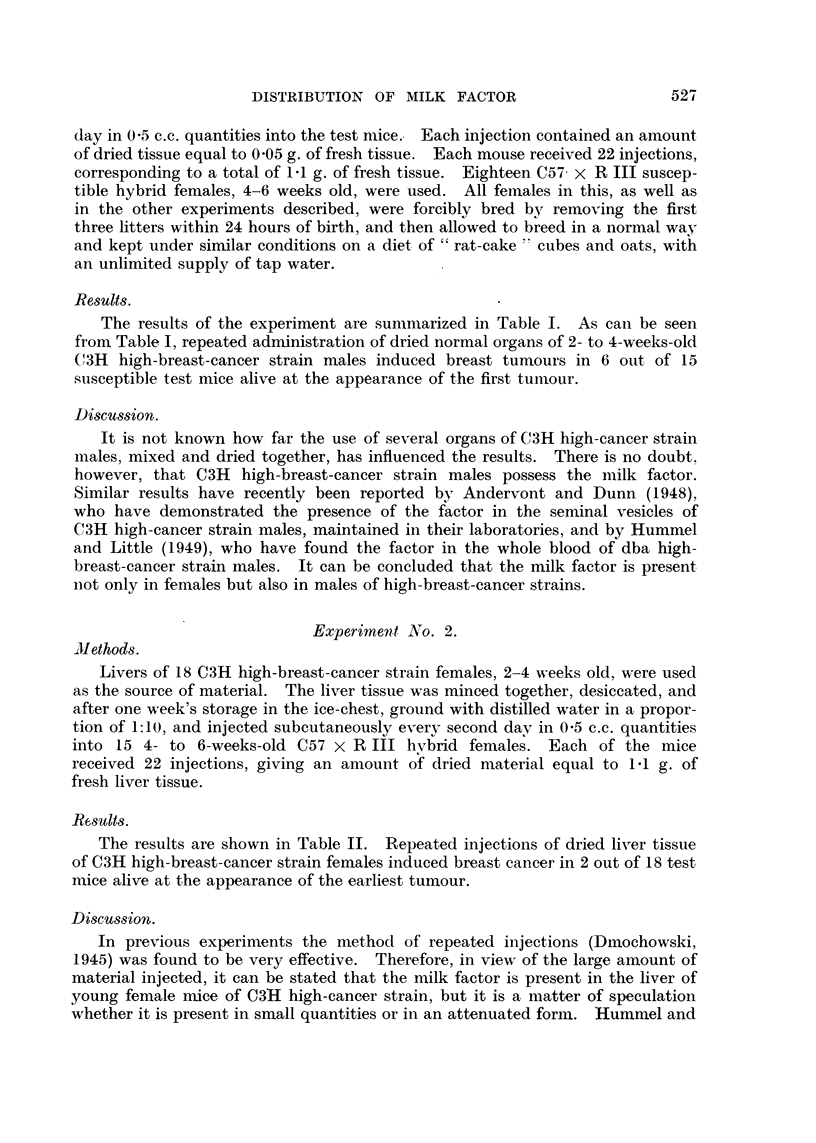

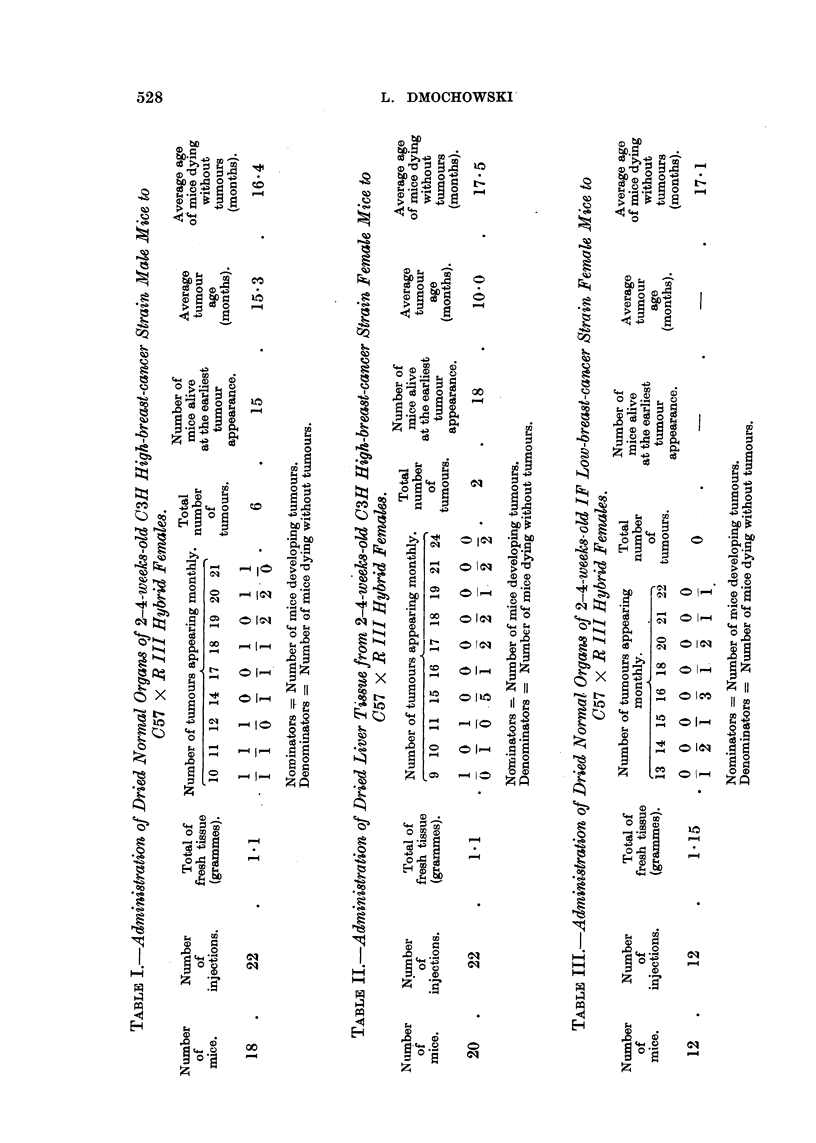

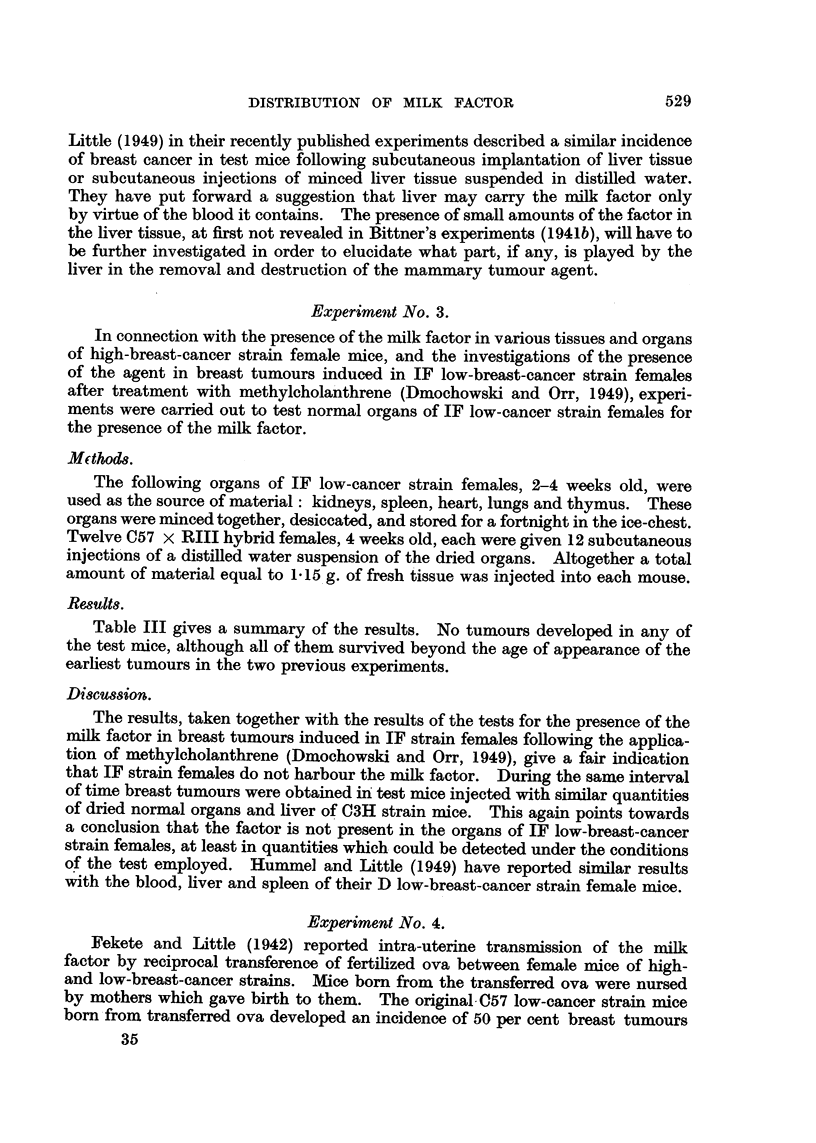

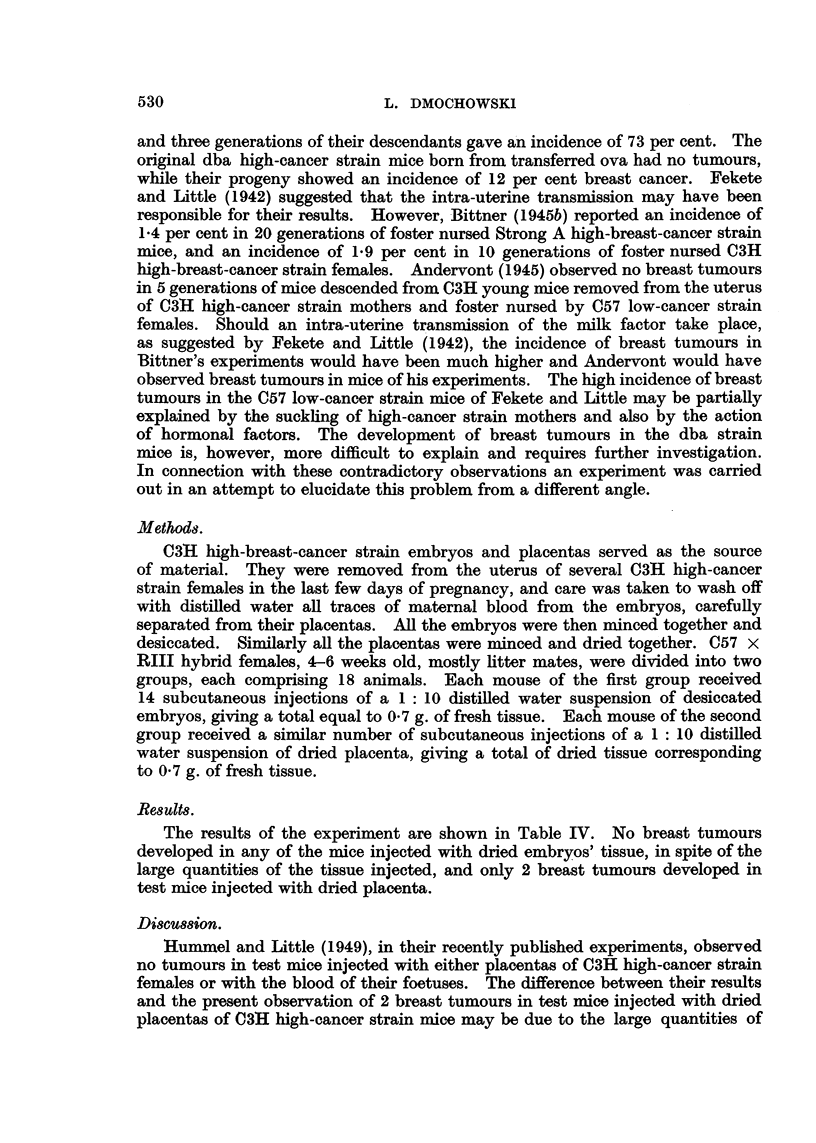

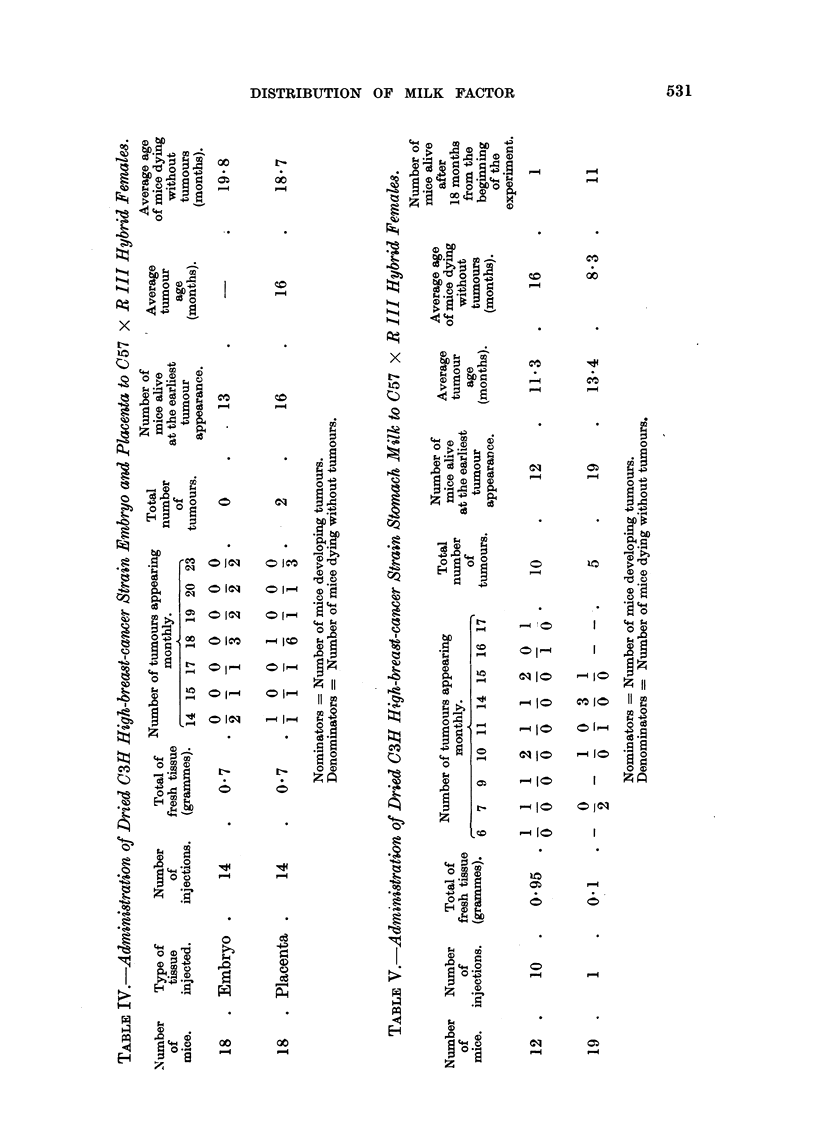

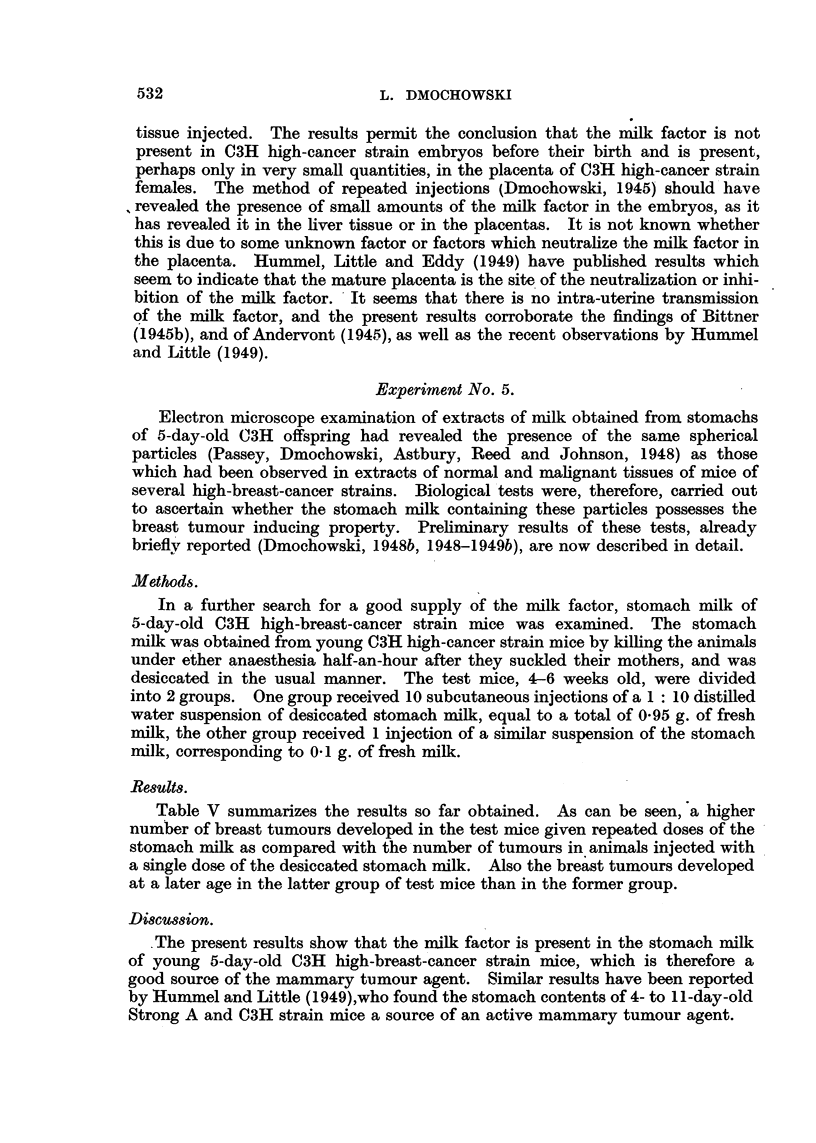

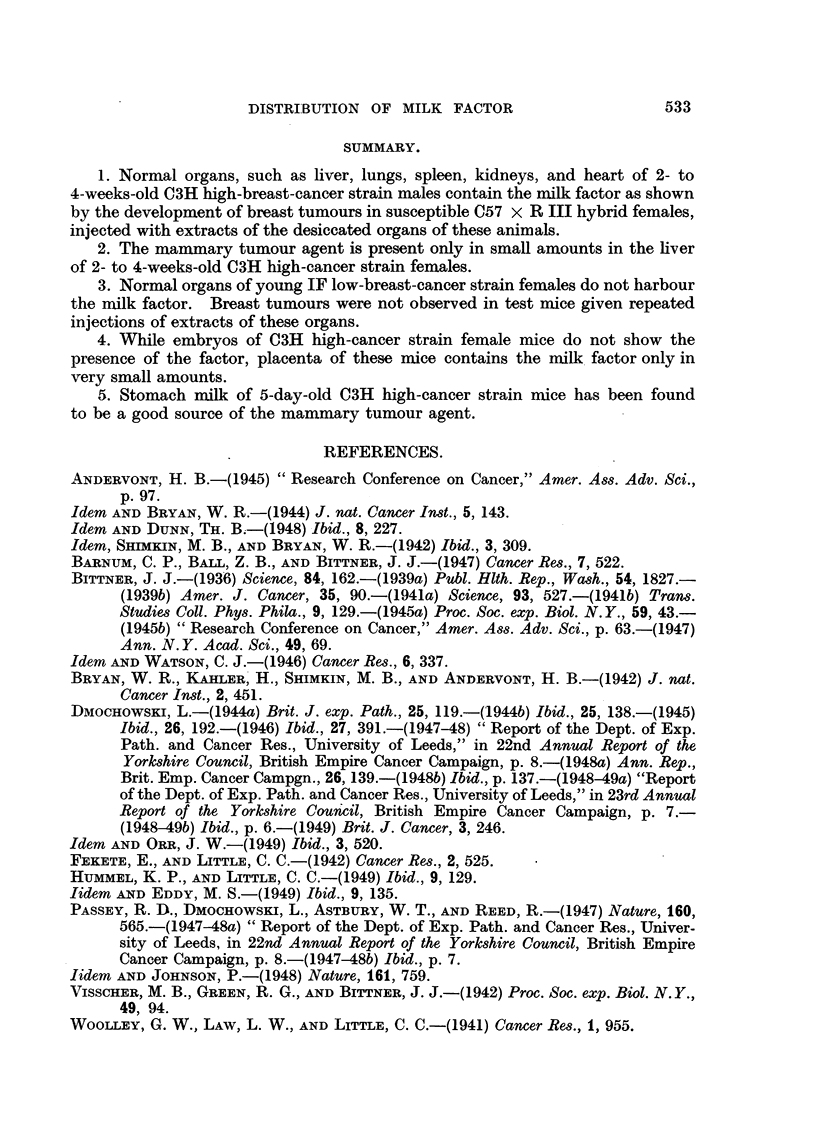


## References

[OCR_00719] Bittner J. J. (1936). SOME POSSIBLE EFFECTS OF NURSING ON THE MAMMARY GLAND TUMOR INCIDENCE IN MICE.. Science.

[OCR_00741] DMOCHOWSKI L., ORR J. W. (1949). Chemically induced breast tumours and the mammary tumour agent.. Br J Cancer.

